# Coping in healthcare personnel: certain coping strategies as predictors of anxiety and depression

**DOI:** 10.1192/bjo.2026.12020

**Published:** 2026-06-15

**Authors:** Paula García-Barja, Beatriz Talavera-Velasco, Daniel Vázquez-Estévez, Lourdes Luceño-Moreno

**Affiliations:** Department of Social and Work Psychology and Individual Differences, Complutense University of Madrid, Madrid, Spain; Division of Psychology, C.E.S. Cardenal Cisneros, Madrid, Spain

**Keywords:** Anxiety, depression, coping strategies, healthcare professionals

## Abstract

**Background:**

Healthcare personnel exhibit higher levels of anxiety and depression, with differences in the use of coping strategies to manage stressful situations.

**Aims:**

To assess which coping strategies, sociodemographic factors and job-related variables predict anxiety and/or depression among healthcare professionals.

**Method:**

A total of 744 participants, including physicians, nurses and nursing assistants were involved in a cross-sectional study.

**Results:**

The ordinary least squares estimator was used to estimate the parameters. The results identified negative self-focus (*β* = 0.42, *β* = 0.45, *p* < 0.001), positive reappraisal (*β* = −0.20, *β* = −0.30, *p* < 0.001) and open emotional expression (*β* = 0.12, *p* < 0.001; *β* = 0.08, *p* < 0.01), as coping strategies significantly associated with anxiety and depression. Additionally, seeking social support was related to depressive symptoms (*β* = −0.02, *p* < 0.01), but not of anxiety. Interestingly, avoidant coping was associated with lower levels of both anxiety and depression (*β* = −0.14, *p* < 0.001). The absence of family responsibilities was associated with lower levels of anxiety and depression (*β* = −0.11, *β* = −0.10, *p* < 0.001). Being male was linked to lower anxiety levels (*β* = −0.11, *p* < 0.001), while being female was associated with greater depressive symptoms (*β* = 0.09, *p* < 0.01). Holding the position of nursing assistant was identified as a variable associated with anxiety and depression (*β* = 0.08, *p* < 0.05; *β* = 0.06, *p* < 0.05).

**Conclusions:**

These results are essential for tailoring interventions aimed at occupational health.

Healthcare professionals face high workloads, elevated emotional tension, extended working hours and limited availability of free time for family and social relationships.^1^ Continuous exposure to adverse working conditions can lead, over time, to symptoms associated with psychological disorders such as anxiety and depression.^2^ These symptoms are more prevalent among healthcare workers than in the general population. Some authors reported a prevalence of 44.6% for anxiety and 50.4% for depressive symptoms.^3^ Similarly, other research has found comparable prevalence rates after analysing 14 studies involving 7780 healthcare workers.^4^ During the COVID-19 period anxiety affected 3–30% of the general non-healthcare population, compared with 32–72% among healthcare professionals.^5^ Regarding depressive symptoms, prevalence in the general population ranged from 6 to 57%, whereas among healthcare professionals, it ranged from 27 to 61%.^5^ Scientific literature has identified several factors associated with anxiety and depression among this professional group, which are explained below. Regarding job roles, nursing assistants have been reported to have the highest prevalence of anxiety and depression compared with physicians and nurses.^6^ However, other authors suggest that nurses experience higher levels of stress, anxiety and depression than other healthcare professionals.^7^ In terms of age, younger nurses and physicians tend to report higher rates of anxiety and depression, particularly those working on the front line.^8^ Conversely, healthcare workers over the age of 50 report fewer symptoms of anxiety and depression.^9^ Other associated variables include gender and educational level. Being female has consistently been linked to higher levels of anxiety and depression among healthcare professionals.^10^ Lower levels of education have also been associated with higher levels of anxiety and depression in this population.^11^ However, some studies suggest that healthcare professionals with a university degree or higher may be more likely to experience stress, anxiety and depression than those with lower educational attainment.^12^ The emergence and severity of anxiety and depression symptoms vary across individuals. Evidence suggests that the way people cope with adverse situations has a significant impact on their quality of life, and the use of maladaptive coping strategies may be associated with stress, anxiety and depression.^13^ Other theoretical models define coping strategies as cognitive and behavioural efforts aimed at managing internal or external demands that exceed an individual’s resources.^14^ Both internal factors (such as coping strategies and personality traits) and external factors (such as available resources and social support) can positively or negatively modulate the impact of stressors. Coping is considered one of the primary defences for protecting workers mental health.^15^ Indeed, during the COVID-19 pandemic, coping strategies played a crucial role in managing workplace stress and emergency situations.^16^ There are eight coping strategies categorised into two broad dimensions: problem-focused and emotion-focused strategies. Problem-focused strategies aim to modify or change the situation and include: (a) confrontation, in which the individual actively attempts to change the situation, and (b) planning. Emotion-focused strategies aim to reduce the emotional distress caused by a stressful situation and include: (c) acceptance, which involves acknowledging the situation and one’s responsibility in it; (d) self-control, referring to the regulation of one’s emotions and behaviours; (e) positive reappraisal, or reinterpreting a stressful situation in a more positive light; (f) distancing, used to psychologically detach from the conflict; (g) escape–avoidance, encompassing cognitive and behavioural attempts to avoid the problem; and (h) seeking social support, which spans both dimensions.^14^ On their behalf, other authors identified seven coping strategies, distinguishing between rational strategies: problem-solving focus, positive reappraisal and seeking social support and emotion-centred strategies, which include negative self-focus, open emotional expression, religious coping and avoidance.^17^ Adaptive coping strategies are those that reduce the impact of stressors and enhance well-being, leading to improved stress management and better overall performance among healthcare professionals.^18^ Adaptive strategies include active coping, planning, suppression of competing activities, restraint, positive reinterpretation and seeking social support. Conversely, maladaptive or dysfunctional coping strategies may provide short-term relief but fail to address the underlying stressor and may even reinforce it in the long term. These include avoidance, rumination, negative self-focus and open emotional expression.^19^

The use of adaptive coping strategies by healthcare professionals is associated with improved patient care, greater safety and reduced healthcare costs.^20^ Some authors emphasised that problem-focused strategies (e.g. active coping, planning, instrumental support, restraint, suppression of competing activities) and emotion-focused strategies (e.g. positive reappraisal, emotional support, acceptance, religious coping, humour) are beneficial and linked to a higher quality of life.^21^ In contrast, dysfunctional coping strategies (e.g. denial, substance use, behavioural and emotional disengagement and venting) have a negative impact on healthcare professionals. Indeed, maladaptive strategies such as avoidance and escape have been shown to increase mental health problems, while strategies such as seeking social support, maintaining a positive attitude, fostering self-efficacy and engaging in problem-solving are associated with better health outcomes and lower stress levels.^16^ The primary aim of this study is to evaluate which coping strategies predict anxiety and/or depression in healthcare professionals. Additionally, it seeks to examine the relationship between sociodemographic, work-related variables and coping strategies with anxiety and depression, with the goal of determining which variables and strategies are the most significant predictors. The main hypotheses are as follows: (a) the use of maladaptive coping strategies (such as negative self-focus, open emotional expression, or avoidance coping) will predict higher levels of anxiety and depression among healthcare professionals; (b) being female and/or younger will be associated with higher levels of anxiety and depression; (c) lower educational levels will be associated with more symptoms of anxiety and depression; and (d) professionals working as nursing assistants will report higher levels of anxiety and depression.

## Method

### Participants

A total of 744 healthcare professionals participated in the study. Of these, 626 (84.1%) were women and 118 (15.9%) were men. The mean age was 44.96 years (s.d. = 11.49), with participants ranging from 21 to 67 years of age. A total of 637 participants (85.6%) were employed in the Community of Madrid, while 107 (14.4%) worked in other regions of Spain. Regarding professional roles, 221 participants (29.7%) were physicians, 243 (32.7%) were nurses or midwives, and 280 (37.6%) were nursing assistants. In terms of marital status, 381 (51.2%) were married, 84 (11.3%) were separated, divorced, or widowed, and 279 (37.5%) were single. Additional demographic and occupational characteristics of the sample are presented in Table 1.

**Table 1:** Association between demographic variables and workers with anxiety and depression (*n* = 744) Table 1 long description.

Variable	Descriptive	Anxiety			Depression		
	*N* (%)	*R* ^2^	*B* (beta)	95% CI	*R* ^2^	*B* (beta)	95% CI
Gender							
Male	118 (15.9)	0.037	−2.285 (−0.192)***	−3.127, −1.442	0.018	−1.490 (−0.135)***	−2.278, −7.02
Female	626 (84.1)						
Age		0.000	0.002 (0.006)	−0.025, 0.030	0.000	−0.001 (−0.002)	−0.026, 0.025
Educational level							
Primary education or high school	47 (6.3)	0.035	1.517 (0.085)*	0.198, 2.835	0.019	0.342 (0.021)	−0.890, 1.573
Medium-level qualification or vocational training	207 (27.8)		1.421 (0.146)***	0.664, 2.179		1.188 (0.132)***	0.480, 1.895
Degree or bachelor’s degree	305 (41)						
Master’s degree or doctorate	185 (24.9)		−0.563 (−0.056)	−1.347, 0.224		−0.173 (−0.019)	−0.906, 0.559
Family responsibilities							
Yes	465 (62.5)				0.008		
No	279 (37.5)	0.003	−0.525 (−0.058)	−1.172, 0.121		−0.729 (0.305)*	−1.327, −0.131
Marital status							
Married	381 (51.2)						
Separated, divorced or widowed	84 (11.3)	0.004	0.341 (0.025)	−0.689, 1.372	0.001	0.278 (0.022)	−0.678, 1.234
Single	279 (37.5)		−0.435 (−0.048)	−1.108, 0.238		−0.151 (−0.018)	−0.776, 0.474

*p < 0.05; ***p < 0.001.

### Instruments

#### Sociodemographic and occupational variables

An ad hoc questionnaire was administered to collect data on the following variables: gender, age, marital status, educational level, family responsibilities, current Autonomous Community of employment, job position, type of healthcare centre, whether or not the participant works in an intensive care unit (ICU), work schedule, if the professional does on-call work and type of employment contract.

#### Anxiety and depression

The Spanish adaptation of the Hospital Anxiety and Depression Scale (HADS) was used.^22^ This instrument consists of 14 items assessing 2 dimensions: anxiety and depression, with 7 items for each. Responses are rated on a 4-point Likert scale ranging from 0 to 3, yielding total scores from 0 to 21 for each subscale. The scale has demonstrated adequate psychometric properties in terms of validity (two-factor structure) and reliability, with Cronbach’s α coefficients of 0.77 for the anxiety subscale and 0.71 for the depression subscale.^22^ These Cronbach’s α indices are considered adequate.^23^ This questionnaire has been designed for use in hospital contexts, making it suitable for use with the healthcare professionals being evaluated.^24^

#### Coping strategies

The Coping with Stress Questionnaire^17^ was employed. This instrument includes 42 items that describe typical ways of thinking and acting when facing stressful or problematic life situations. It assesses seven core coping styles, each represented by six items: problem-focused coping (PFS), negative self-focus (NSF), positive reappraisal, open emotional expression (OEE), avoidance coping, seeking social support (SSS), and religious coping (RLG). Responses are given on a five-point Likert scale ranging from 0 (never) to 4 (almost always). Each subscale yields a score ranging from 0 to 24, with higher scores indicating greater use of that coping strategy. The questionnaire has shown a good fit to a seven-factor structure representing the seven coping styles, with reliability indices ranging from 0.64 to 0.92.^17^

### Procedure

A cross-sectional correlational design was used. The authors assert that all procedures contributing to this work comply with the ethical standards of the relevant national and institutional committees on human experimentation and with the Helsinki Declaration of 1975, as revised in 2013. All procedures involving human subjects were approved by the Research Ethics Committee of the Complutense University of Madrid (Ref.: CE-20220519-05-SOC). The target population consisted of healthcare professionals working in hospitals and primary care centres within the Spanish public healthcare system. Data were collected through a Google Forms questionnaire that included sociodemographic and work-related variables, along with the rest of the study instruments. Written consent was obtained. The worker had to indicate that he/she had read the informed consent form and agreed to participate in the research. The consent appeared at the beginning of the questionnaire, before participants viewed the questions. The survey was distributed through the administrative staff of various hospitals and primary care centres across different Autonomous Communities of Spain. The study procedure was approved by the Ethics Committee prior to its implementation.

### Data analysis

Data analyses were conducted using IBM SPSS Statistics for Windows, version 26.0 (IBM Corp., Armonk, NY, USA; https://www.ibm.com/support/pages/downloading-ibm-spss-statistics-26-transition-extended-support-30-sep-2025). Descriptive statistics (frequencies, mean and s.d.) were calculated for anxiety, depression and coping strategies. Linear regression equations were used to assess the relationship between each sociodemographic (variables in Table 1), occupational (variables in Table 2) and coping strategy variable with anxiety and depression scores. The coefficient of determination (*R* ^2^) and the standardised beta coefficient (*β*), along with their corresponding CI, were reported. To facilitate the analysis of categorical variables, these were transformed into dummy variables, which were used in the analyses as predictor variables. Finally, multivariate linear regression models were applied to identify which sociodemographic, occupational and coping strategy variables (considered together) were associated with anxiety and depression. The models were estimated using the least squares method, employing a forward stepwise selection procedure. Statistical significance was set at *p* < 0.05. The forward method was used to assess the predictive power of variables known in previous literature to be relevant in the prediction of anxiety and depression.

**Table 2:** Association between job variables with anxiety and depression (*n* = 744) Table 2 long description.

Variable	Descriptive	Anxiety			Depression		
	*N* (%)	*R* ^2^	*B* (beta)	95% CI	*R* ^2^	*B* (beta)	95% CI
Job position							
Physician	221 (29.7)	0.037	−1.012 (−0.106)*	−1.793, −0.231	0.020	−0.617 (−0.070)	−1.347, 0.113
Nurse or midwife	243 (32.7)						
Nursing assistant	280 (37.6)		1.034 (0.357)*	0.298, 1.771		0.756 (0.090)*	0.068, 1.445
On-call work							
Yes	254 (34.1)						
No	490 (65.9)	0.002	0.393 (0.043)	−0.268, 1.053	0.000	0.099 (0.012)	−0.514, 0.712
Work in ICU							
Yes	655 (88)						
No	89 (12)	0.000	−0.222 (−0.017)	−1.188, 0.745	0.001	−0.470 (−0.038)	−1.364, 0.425
Type of work day							
Part-time	62 (8.3)						
Full	682 (91.7)	0.003	−0.834 (−0.053)	−1.967, 0.299	0.003	−0.817 (−0.056)	−1.867, 0.233
Contract type							
Permanent status	199 (26.7)		−0.461 (−0.047)	−1.307, 0.385		−0.122 (−0.013)	−0.906, 0.663)
Permanent staff status	207 (27.8)						
Interim or long-term substitute	198 (26.6)	0.011	0.294 (0.030)	−0.554, 1.141	.011	0.708 (0.078)	−0.077, 1.494
Temporary or short term	90 (12.1)		−0.959 (0.548)	−2.035, 0.117		−0.429 (−0.035)	−1.426, 0.568
Training contract	50 (6.7)		−1.092 (−0.063)	−2.435, 0.251		−0.533 (−0.033)	−1.778, 0.711
Autonomous Community							
Community of Madrid	637 (85.6)						
Other	107 (14.4)	0.003	−0.695 (−0.056)	−1.587, 0.197	0.007	−0.971 (−0.085)*	−1.796, −0.146
Work centre							
Hospital	511 (68.7)						
Primary care	103 (13.8)	0.005	−0.540 (−0.043)	−1.462, 0.382	0.000	−0.216 (−0.019)	−1.073, 0.640
Other centre	130 (17.5)		0.529 (0.046)	−.310, 1.368		−0.077 (−0.007)	−0.856, 0.702

ICU, intensive care unit. *p < 0.05.

## Results

Regarding sociodemographic variables, being male was negatively associated with both anxiety and depression. Concerning educational level, having completed only primary education or high school was positively associated with anxiety, whereas having a medium-level qualification or vocational training degree was positively associated with both anxiety and depression. Additionally, not having family responsibilities was negatively associated with depression. Being a man is negatively associated with both anxiety and depression. The model was not statistically significant for age (see Table 1).

In terms of work-related variables, being a physician was negatively associated with anxiety, while being a nursing assistant was positively associated with both anxiety and depression. Furthermore, working in a region other than the Community of Madrid was negatively associated with depression (see Table 2).

With respect to coping strategies, problem-focused coping, positive reappraisal and seeking social support were negatively and significantly associated with both anxiety and depression, although seeking social support had a very small effect size.

In contrast, negative self-focus and open emotional expression were positively and significantly associated with both anxiety and depression. Additionally, avoidance coping and religious coping strategies were negatively and significantly associated only with depression (see Table 3).

**Table 3:** Association between coping strategies with anxiety and depression (*n* = 744) Table 3 long description.

Variable	Anxiety			Depression		
	*R* ^2^	*B* (beta)	95% CI	*R* ^2^	*B* (beta)	95% CI
CSQ_PSF	0.104	−0.306 (−0.322)***	−0.371, −0.241	0.199	−0.393 (−0.446)***	−0.450, −0.336
CSQ_NSF	0.304	0.644 (0.036)***	0.574, 0.714	0.374	0.662 (0.612)***	0.600, 0.724
CSQ_PR	0.153	−0.441 (−0.391)***	−0.516, −0.366	0.277	−0.550 (−0.527)***	−0.614, −0.486
CSQ_OEE	0.077	0.372 (0.278)***	0.280, 0.465	0.058	0.300 (0.242)***	0.214, 0.387
CSQ_AC	0.003	−0.067 (−0.056)	−0.154, 0.020	0.021	−0.163 (−0.146)***	−0.243, −0.083
CSQ_SSS	0.012	−0.078 (−0.110)**	−0.129, −0.027	0.045	−0.139 (−0.212)***	−0.185, −0.093
CSQ_RLG	0.001	−0.029 (−0.036)	−0.087, 0.029	0.006	−0.058 (−0.078)*	−0.112, −0.005

CSQ_PSF, problem-solving focus; CSQ_NSF, negative self-focus; CSQ_PR, positive reappraisal; CSQ_OEE, open emotional expression; CSQ_AC, avoidant coping; CSQ_SSS, seeking social support; CSQ_RLG, religious coping. *p < 0.05; **p < 0.01; ***p < 0.001.

Results related to internal consistency and correlations between factors are presented in Table 4. The anxiety scale showed a strong positive correlation with the negative self-focus coping strategy. The depression scale was strongly and positively correlated with negative self-focus and moderately and negatively correlated with positive reappraisal. Problem-solving focus was strongly and positively correlated with positive reappraisal and moderately and positively correlated with seeking social support (see Table 4).

**Table 4:** Correlation matrix (*n* = 744) Table 4 long description.

Variable	1	2	3	4	5	6	7	8	9
HADS_A	–								
HADS_D	0.744***	–							
CSQ_PSF	−0.322***	−0.446***	–						
CSQ_NSF	0.551***	0.612***	−0.392***	–					
CSQ_PR	−0.391***	−0.527***	0.633***	−0.382***	–				
CSQ_OEE	0.278***	0.242***	−0.069	0.307***	−0.108**	–			
CSQ_AC	−0.056	−0.146***	0.250***	−0.008	0.367***	0.027	–		
CSQ_SSS	−0.110**	−0.212***	0.440***	−0.085*	0.351***	0.250***	0.241***	–	
CSQ_RLG	−0.036	−0.078*	0.220***	−0.041	0.183***	0.006	0.024	0.180***	–
*M*	9.56	6.54	14.9	8.91	14.35	7.30	11.53	12.11	3.77
*SD*	4.35	4.03	4.58	3.72	3.86	3.25	3.60	6.15	5.42
α	0.875	0.861	0.871	0.691	0.754	0.671	0.610	0.938	0.930

HADS_A, anxiety; HADS_D, depression; CSQ_PSF, problem-solving focus; CSQ_NSF, negative self-focus; CSQ_PR, positive reappraisal; CSQ_OEE, open emotional expression; CSQ_AC, avoidant coping; CSQ_SSS, seeking social support; CSQ_RLG, religious coping. *p < 0.05; **p < 0.01; ***p < 0.001.

Normality was checked using standardised residual graphs, and both models showed normality fit. Independence was checked using the Durbin–Watson test. Both statistics were close to 2 (HADS A: 1.90; HADS D: 1.92), showing independence between prediction errors. Finally, multicollinearity was checked using Tolerance (TOL) and Variance Inflation Factor (VIF) indicators. For both models, Tolerance indicators were greater than 0.2 and VIF indicators were lower than 12, showing no presence of multicollinearity between predictors. Multicollinearity was assessed using the VIF and TOL statistics; there are no multicollinearity issues in the model.

The regression model for anxiety was statistically significant and explained 40.3% of the variance (*F*(7, 658) = 63.509, *p* < 0.001). The model for depression was also significant, explaining 50.1% of the variance (*F*(7, 658) = 94.315, *p* < 0.001). Variables common to both models included the coping strategies of negative self-focus, positive reappraisal, open emotional expression and the professional role of nursing assistant. Variables positively associated with anxiety included negative self-focus, open emotional expression and being a nursing assistant. Variables negatively associated with anxiety were positive reappraisal, not having family responsibilities and being male. For depression, the variables positively associated were negative self-focus, open emotional expression, being female and being a nursing assistant. In contrast, depression was negatively associated with positive reappraisal, seeking social support and not having family responsibilities (see Table 5).

**Table 5:** Regression model for anxiety and depression (*n* = 744) Table 5 long description.

Variable	Anxiety		Depression	
	*B* (beta)	95% CI	*B* (beta)	95% CI
CSQ_NSF	0.495 (0.424)***	0.417, 0.573	0.489 (0.451)***	(0.422, 0.556)
CSQ_PR	−0.231 (−0.204)***	−0.304, −0.158	−3.18 (−0.303)***	(−0.384, −0.253)
CSQ_OEE	0.160 (0.121)***	0.076, 0.244	0.105 (0.086)**	(0.032, 0.179)
CSQ_SSS			−0.055 (−0.021)**	(−0.096, −0.014)
Male	−1.418 (−0.117)***	−2.166, −0.671		
Female			1.061 (0.094)**	(0.423, 1.700)
Nursing assistant	0.773 (0.086)*	0.181, 1.364	0.526 (0.063)*	(0.058, 0.994)
Family responsibilities (No)	−1.013 (−0.111)***	−1.564, −0.461	−0.861 (−0.102)***	(−1.329, −0.394)

CSQ_NSF, negative self-focus; CSQ_PR, positive reappraisal; CSQ_OEE, open emotional expression; CSQ_SSS, seeking social support. *p < 0.05; **p < 0.01; ***p < 0.001.

## Discussion

The main objective of this study was to evaluate which coping strategies predict anxiety and/or depression among healthcare professionals. Additionally, the study examined which sociodemographic and occupational variables are associated with anxiety and depression, aiming to determine which variables and coping strategies have the most significant predicting power for these psychological outcomes. The results identified the following coping strategies as predictors of both anxiety and depression: negative self-focus, positive reappraisal and open emotional expression. Negative self-focus and open emotional expression were associated with higher levels of anxiety and depression, while positive reappraisal was associated with lower levels of these symptoms. Additionally, seeking social support emerged as a predictor of depressive symptoms only, according to our model. Other studies have also emphasised the usefulness of social support in alleviating work-related stress among healthcare workers.^25^ Research has shown that social support plays a crucial role in this professional group, as relationships with family and friends can buffer the effects of depressive symptoms and feelings of loneliness in the workplace.^26^ Regarding open emotional expression, previous research has emphasised the importance of reducing impulsive and uncontrolled emotional expression, as it is associated with depressive states and a greater likelihood of engaging in risk-related or dysregulated behaviours.^27^ However, it should be noted that emotional expression per se is not inherently maladaptive, as its effects may vary depending on the degree of regulation and the cultural or contextual framework.^28^ In contrast, positive reappraisal has been shown to be an effective coping strategy, especially in the face of traumatic situations. It fosters optimism, minimises catastrophising and encourages acceptance, thereby helping individuals adapt to stress, grief and sadness. This strategy has also been linked to better functionality, reduced presenteeism and even greater effectiveness when combined with social support.^29^ Therefore, the first hypothesis of this study is partially supported. Maladaptive strategies such as negative self-focus and open emotional expression were indeed associated with higher levels of anxiety and depression. However, only negative self-focus emerged as a predictor of both conditions, while open emotional expression was predictive of depression alone.

Interestingly, avoidant coping in our study was associated with lower levels of both anxiety and depression. This finding is particularly relevant, as individuals often resort to avoidance when they perceive they lack the resources to face environmental threats. This strategy typically includes efforts to withdraw from the stressful situation, avoid problems, seek emotional release, or disengage behaviourally or cognitively.^30^ In this cross-sectional study, the inverse association between avoidance and depression could be explained by the regulatory function that certain avoidance strategies perform in emotionally demanding contexts such as healthcare, which is characterised by high chronic emotional exposure. In these environments, avoidance can reduce repetitive cognitive processing and rumination, processes closely linked to the development and maintenance of depressive symptoms.^31^ In this sense, the impact of avoidance on depression would depend on the context and the time frame evaluated, which could explain the results observed. In any case, these contradictory findings suggest the need for further research to explore and clarify these relationships.

Regarding sociodemographic predictors, both the absence of family responsibilities and gender were associated with anxiety and depression. Specifically, participants without family responsibilities reported fewer symptoms. Conversely, other studies indicate that healthcare workers with caregiving duties, particularly nurses, and especially during the pandemic, showed higher levels of stress, anxiety and depression.^32^ These results may be explained by heightened concern about infecting loved ones and the associated sense of responsibility. Being male was associated with lower levels of anxiety, while being female was linked to higher depressive symptoms. Although some studies have reported mixed findings on this matter,^33^ others confirm gender differences in the prevalence of anxiety and depression among healthcare professionals, with women showing higher symptom levels.^34^ However, it is important to note that the observed gender differences may be partially explained by structural factors, such as gendered workload allocation, or workplace discrimination, rather than intrinsic differences.^35^ Therefore, our second hypothesis is partially supported: being female did predict depression, although no significant relationship with anxiety was observed in our model. In contrast, age was not associated with either anxiety or depression and did not emerge as a predictor. Concerning the third hypothesis, a relationship was found between lower educational levels and increased symptoms of anxiety and depression. However, educational level did not appear as a predictor in the final model. Some authors argue that lower educational attainment is associated with positions involving high emotional strain,^36^ whereas higher education may lead to better employment opportunities characterised by greater autonomy and professional development, thereby reducing psychological distress.^37^ It is possible that these gender and occupational differences reflect not only individual characteristics but also organisational and structural factors – such as workload, autonomy and job insecurity – which have been shown to be linked to the psychological well-being of healthcare workers.^38^ Future studies should consider these contextual relationships to better understand mental health among healthcare professionals.

Regarding work-related variables, the role of nursing assistant emerged as a predictor of both anxiety and depression, supporting our fourth hypothesis. Previous research has shown that professionals in lower-level positions tend to report higher levels of anxiety and depression,^39^ possibly due to reduced decision-making autonomy or more precarious working conditions compared with those in higher-ranking roles.^40^ The findings of this study highlight the importance of incorporating coping strategies that predict anxiety and depression into prevention and intervention programmes for healthcare professionals. Specifically, this research supports promoting positive reappraisal and reducing the use of maladaptive strategies such as negative self-focus and open emotional expression. Furthermore, preventive strategies aimed at reducing depressive symptoms should include the promotion of social support. This study also emphasises the importance of considering key sociodemographic variables – such as family responsibilities and gender – when designing tailored interventions. This means that actions can be tailored to specific groups of workers (women, people with family responsibilities), which can improve the specificity and effectiveness of such interventions. Special attention should also be given to nursing assistants, as this group appears particularly vulnerable to anxious and depressive symptoms. It is recommended that these coping strategies be incorporated into emotional psychoeducation interventions for healthcare personnel, such as resilience workshops, group problem-solving, cognitive–behavioural training and mindfulness which have been shown to be effective in improving psychological well-being.

Finally, several limitations should be noted. The sample was incidental and non-randomised. It would be beneficial to increase the number of participants across job categories to assess potential differences (e.g. by region, healthcare setting, etc.). On the other hand, most of the sample is represented by women, people who work in Madrid, or nursing assistants, which may limit the generalisability of the results. Also, recruitment via Google Forms may have introduced self-selection bias, particularly if those experiencing higher distress were more likely to respond. Some of the scales in the coping questionnaire have Cronbach’s α coefficients below 0.70, so interpretations made for these scales should be treated with caution. The results for the seeking social support coping strategy should be confirmed in other studies with healthcare personnel, as the effect size for this factor is low in this study. Another limitation of the study is that there is a high correlation between the problem-solving orientation scales and the positive reappraisal scale. However, no multicollinearity issues were observed. Furthermore, this study was unable to employ other statistical techniques that require a larger sample size. For example, it was not possible to develop an integrated model based on structural equation modelling or an approach based on machine learning, as this would have required a larger sample. Given the cross-sectional nature of the study, no conclusions about causality or changes over time can be drawn. Future research should explore the role of coping strategies in longitudinal studies and evaluate their effectiveness through targeted interventions to reduce anxiety and depression symptoms. Moreover, linking these outcomes with organisational indicators such as absenteeism, turnover intentions, staff rotation and medical leave could provide valuable insights for optimising workforce costs in public healthcare systems.

## Data Availability

The data that support the findings of this study are openly available in Figshare at http://doi.org/10.6084/m9.figshare.29489147.

## Table 1 Long description

The table presents data on the association between demographic variables and workers with anxiety and depression among 744 healthcare professionals. It includes variables such as gender, age, educational level, family responsibilities, and marital status. The table is divided into three main sections: Descriptive, Anxiety, and Depression. The Descriptive section provides the number and percentage of participants for each variable. The Anxiety section includes R-squared values, B (beta) coefficients, and 95% confidence intervals. The Depression section similarly includes R-squared values, B (beta) coefficients, and 95% confidence intervals. Notable trends include a higher percentage of female participants (84.1%) compared to male participants (15.9%), and a significant association between gender and both anxiety and depression. Educational level also shows varying associations with anxiety and depression, with higher education levels generally showing lower B (beta) coefficients. Family responsibilities and marital status are additional variables analyzed for their association with anxiety and depression.

Navigate back to Table 1.

## Table 2 Long description

The table presents data on the association between various job variables and levels of anxiety and depression among 744 individuals. It includes descriptive statistics, R-squared values, beta coefficients, and 95 percent confidence intervals for each variable. The variables analyzed include job position, on-call work, work in ICU, type of workday, contract type, autonomous community, and work center. Notable findings include significant associations for certain job positions, contract types, and work centers with anxiety and depression. For instance, nursing assistants show a positive association with anxiety, while permanent staff status shows a negative association with depression.

Navigate back to Table 2.

## Table 3 Long description

The table presents data on the association between various coping strategies and levels of anxiety and depression. It includes variables such as problem-solving focus, negative self-focus, positive reappraisal, emotional expression, acceptance, social support seeking, and religious coping. For each variable, the table provides R squared values, beta coefficients, and ninety-five percentage confidence intervals for both anxiety and depression. Notable trends include negative associations between problem-solving focus and both anxiety and depression, and positive associations between negative self-focus and both conditions. Avoidance coping and religious coping strategies show significant negative associations only with depression.

Navigate back to Table 3.

## Table 4 Long description

The table presents a correlation matrix with 744 participants, showing the relationships between different psychological factors and coping strategies. It includes nine variables: HADS_A, HADS_D, CSQ_PSF, CSQ_NSF, CSQ_PR, CSQ_QEE, CSQ_AC, CSQ_SSS, and CSQ_RLG. The anxiety scale (HADS_A) shows a strong positive correlation with the negative self-focus coping strategy (CSQ_NSF). The depression scale (HADS_D) is strongly and positively correlated with negative self-focus and moderately and negatively correlated with positive reappraisal (CSQ_PR). Problem-solving focus (CSQ_PSF) is strongly and positively correlated with positive reappraisal and moderately and positively correlated with seeking social support (CSQ_SSS). The table also includes mean values (M), standard deviations (SD), and Cronbach’s alpha (α) for each variable. Row 1: HADS_A. Row 2: HADS_D, 0.744, 6.54, 4.35, 0.875. Row 3: CSQ_PSF, -0.322, -0.446, 14.9, 4.58, 0.871. Row 4: CSQ_NSF, 0.551, 0.612, -0.392, 14.58, 3.72, 0.691. Row 5: CSQ_PR, -0.391, -0.527, 0.633, -0.382, 14.35, 3.86, 0.754. Row 6: CSQ_QEE, 0.278, 0.242, -0.069, 0.307, -0.108, 7.30, 3.25, 0.671. Row 7: CSQ_AC, -0.056, -0.146, 0.250, -0.008, 0.367, 0.027, 11.53, 3.60, 0.610. Row 8: CSQ_SSS, -0.110, -0.212, 0.440, -0.085, 0.351, 0.250, 0.241, 12.11, 6.15, 0.938. Row 9: CSQ_RLG, -0.036, -0.078, 0.220, -0.041, 0.183, 0.006, 0.024, 0.180, 3.77, 5.42, 0.930.

Navigate back to Table 4.

## Table 5 Long description

The table presents a regression model for anxiety and depression, including variables such as coping strategies and professional roles. It consists of eight rows and six columns. The columns are labeled Variable, Anxiety B (beta), Anxiety 95% CI, Depression B (beta), and Depression 95% CI. The variables include coping strategies like negative self-focus, positive reappraisal, open emotional expression, and seeking social support, as well as demographic factors like gender and family responsibilities. The table shows beta coefficients and 95% confidence intervals for each variable’s association with anxiety and depression. Notable trends include positive associations of negative self-focus and open emotional expression with both anxiety and depression, while positive reappraisal is negatively associated with both. Being a nursing assistant is positively associated with both anxiety and depression. Gender differences are noted, with being male negatively associated with anxiety and being female positively associated with depression. Not having family responsibilities is negatively associated with both anxiety and depression.

Navigate back to Table 5.

## References

[1] Chen Q , Liang M , Li Y , Guo J , Fei D , Wang L , et al. Mental health care for medical staff in China during the COVID-19 outbreak. Lancet Psychiatry 2020; 7: e15–6. 32085839 10.1016/S2215-0366(20)30078-X PMC7129426

[2] Sun H , Zhang T , Wang X , Wang C , Zhang M , Song H. The occupational burnout among medical staff with high workloads after the COVID-19 and its association with anxiety and depression. Front Public Health 2023; 11: 1–16. 10.3389/fpubh.2023.1270634 PMC10639132 37954047

[3] Lai J , Ma S , Wang Y , Cai Z , Hu J , Wei N , et al. Factors associated with mental health outcomes among health care workers exposed to coronavirus disease 2019. JAMA Netw Open 2020; 3: 1–12. 10.1001/jamanetworkopen.2020.3976 PMC7090843 32202646

[4] Sialakis C , Sialaki PA , Frantzana A , Iliadis C , Ouzounakis P , Kourkouta L. Prevalence of anxiety and depression of health care workers during COVID-19 – a systematic review and meta-analysis. Med Pharm Rep 2023; 96: 246–53. 37577023 10.15386/mpr-2579 PMC10419692

[5] Clavero C , Ausín B. Psychological effects of lockdown due to the COVID-19 pandemic in the year 2020: a systematic review. Psicol Conduct 2022; 30: 565–95.

[6] Azoulay E , Cariou A , Bruneel F , Demoule A , Kouatchet A , Reuter D , et al. Symptoms of anxiety, depression, and peritraumatic dissociation in critical care clinicians managing patients with COVID-19. A cross-sectional study. Am J Respir Crit Care Med 2020; 202: 1388–98. 32866409 10.1164/rccm.202006-2568OC PMC7667906

[7] Fageera W , Babtain F , Alzahrani AS , Khrad HM. Lock-down effect on the mental health status of healthcare workers during COVID-19 pandemic. Front Psychiatry 2021; 12: 1–16. 10.3389/fpsyt.2021.683603 PMC8414976 34483984

[8] Vizheh M , Qorbani M , Arzaghi SM , Muhidin S , Javanmard Z , Esmaeili M. The mental health of healthcare workers in the COVID-19 pandemic: a systematic review. J Diabetes Metab Disord 2020; 19: 1967–78. 33134211 10.1007/s40200-020-00643-9 PMC7586202

[9] Gavana M , Papageorgiou DI , Stachteas P , Vlachopoulos N , Pagkozidis I , Angelopoulou P , et al. The psychological impact of COVID-19 pandemic on primary health care professionals in Greece. Psychiatriki 2023; 34: 181–92. 37212800 10.22365/jpsych.2023.008

[10] Cárdaba-García RM , Soto-Cámara R , García-Santa-Basilia N , Matellán-Hernández MP , Onrubia-Baticón H , Martínez-Caballero CM , et al. Impact of the COVID-19-pandemic and perception of self-efficacy on the mental health of out-of-hospital emergency healthcare professionals by modality of care. J Adv Nurs 2024; 80: 3692–704. 38444126 10.1111/jan.16119

[11] Sarvandian S , Hosseinpour S , Hoseinynejad K , Davasaz Irani R , Pakseresht S , Rahimi Z. Mental health status in healthcare workers during COVID-19 pandemic: an online questionnaire study in the southwest Iran. PLOS One 2024; 19: 1–15. 10.1371/journal.pone.0298058 PMC11051651 38669302

[12] Itam MF , Minhat HS , Abd Rahman A , Ibrahim MZ , Shareh Ali SA , Shuhaimi AH. The COVID-19 pandemic related stress and the associated factors among the healthcare workers in Kota Setar District Health Office, Malaysia. PLOS One 2024; 19: 1–9. 10.1371/journal.pone.0301469 PMC11115278 38781199

[13] Wang H , Xia Q , Xiong Z , Li Z , Xiang W , Yuan Y , et al. The psychological distress and coping styles in the early stages of the 2019 coronavirus disease (COVID-19) epidemic in the general mainland Chinese population: a web-based survey. PLOS One 2020; 15: 1–10. 10.1371/journal.pone.0233410 PMC7224553 32407409

[14] Lazarus RS , Folkman S. Estrés y Procesos Cognitivos [Stress and Cognitive Processes]. Martínez Roca, 1986.

[15] Huang L , Lei W , Xu F , Liu H , Yu L. Emotional responses and coping strategies in nurses and nursing students during COVID-19 outbreak: a comparative study. PLOS One 2020; 15: 1–12. 10.1371/journal.pone.0237303 PMC7413410 32764825

[16] Finstad GL , Giorgi G , Lulli LG , Pandolfi C , Foti G , León-Perez JM , et al. Resilience, coping strategies and posttraumatic growth in the workplace following COVID-19: a narrative review on the positive aspects of trauma. Int J Environ Res Public Health 2021; 18: 1–24. 10.3390/ijerph18189453 PMC8468098 34574378

[17] Sandín B , Chorot P. Cuestionario de Afrontamiento del Estrés (CAE): desarrollo y validación preliminar [Coping with Stress Questionnaire (CAE): development and preliminary validation]. Rev Psicopatol Psicol Clin 2003; 8: 39–54.

[18] Abraham LJ , Thom O , Greenslade JH , Wallis M , Johnston AN , Carlström E , et al. Morale, stress and coping strategies of staff working in the emergency department: a comparison of two different-sized departments. Emerg Med Australas 2018; 30: 375–81. 29363265 10.1111/1742-6723.12895

[19] Carver CS , Scheier MF , Weintraub JK. Assessing coping strategies: a theoretically based approach. J Pers Soc Psychol 1989; 56: 267–83. 2926629 10.1037//0022-3514.56.2.267

[20] Marco CA , Broderick K , Smith-Coggins R , Goyal DG , Joldersma KB , Coombs AB. Health and wellness among emergency physicians: results of the 2014 ABEM longitudinal study. Am J Emerg Med 2016; 34: 1715–6. 27321937 10.1016/j.ajem.2016.06.019

[21] Crișan CA , Pop R , Stretea R , Milhem Z , Forray AI. Coping strategies, resilience and quality of life: reaction to the COVID-19 pandemic among Romanian physicians. Hum Resour Health 2024; 22: 1–11. 38715124 10.1186/s12960-024-00909-w PMC11075254

[22] Terol MC , López-Roig S , Rodríguez-Marín J , Martín-Aragón M , Pastor MA , Reig MT. Propiedades psicométricas de la Escala Hospitalaria de Ansiedad y Depresión (HAD) en población española. [Hospital Anxiety and Depression Scale (HAD): psychometric properties in Spanish population]. Ansiedad y Estrés 2007; 13: 163–76.

[23] Revelle W , Zinbarg RE. Coefficients alpha, beta, omega and the glb: comments on Sijtsma. Psychometrika 2009; 74: 145–54.

[24] Bjelland I , Dahl AA , Haug TT , Neckelmann D. The validity of the Hospital Anxiety and Depression Scale. An updated literature review. J Psychosom Res 2002; 52: 69–77. 11832252 10.1016/s0022-3999(01)00296-3

[25] Wang YZ , Xu Y , Ren LZ , Wang Y , Xu YH , Wang YF. The impact of uncertainty stress and social support on the occupational well-being of healthcare professionals during public health emergencies. Work 2025; 80: 908–17. 39973712 10.1177/10519815241290021

[26] Kulari G , Pereira de Castro M. Depressive symptoms, loneliness and social support in healthcare employees: does the source of support matter? J Public Ment Health 2024; 23: 348–56.

[27] Alfonso-Benlliure V , Meléndez Moral JC. Creativity as a ‘vaccine’ for depressed mood: coping and divergent thinking in young adults. An Psicol 2022; 38: 209–18.

[28] Gross JJ , John OP. Individual differences in two emotion regulation processes: implications for affect, relationships, and well-being. J Pers Soc Psychol 2003; 85: 348–62. 12916575 10.1037/0022-3514.85.2.348

[29] Mete B , Demirhindi H , Kahramanoğlu Pİ. , Şahin CK , Tanır F. Medical doctors’ coping strategies with post-earthquake stress and their relationship with presenteeism. Stress Health 2024; 40: 1–7. 10.1002/smi.3352 38084794

[30] Roth S , Cohen LJ. Approach, avoidance, and coping with stress. Am Psychol 1986; 41: 813–9. 3740641 10.1037//0003-066x.41.7.813

[31] Watkins ER. Constructive and unconstructive repetitive thought. Psychol Bull 2008; 134: 163–206. 18298268 10.1037/0033-2909.134.2.163 PMC2672052

[32] Alzailai N , Barriball KL , Alkhatib A , Xyrichis A. Factors that contributed to burnout among intensive care nurses during the COVID-19 pandemic in Saudi Arabia: a constructivist grounded theory. Aust Crit Care 2023; 36: 19–27. 36437164 10.1016/j.aucc.2022.11.002 PMC9643309

[33] Alnazly E , Khraisat OM , Al-Bashaireh AM , Bryant CL. Anxiety, depression, stress, fear and social support during COVID-19 pandemic among Jordanian healthcare workers. PLOS One 2021; 16: 1–22. 10.1371/journal.pone.0247679 PMC7954309 33711026

[34] Brulin E , Lidwall U , Seing I , Nyberg A , Landstad B , Sjöström M , et al. Healthcare in distress: a survey of mental health problems and the role of gender among nurses and physicians in Sweden. J Affect Disord 2023; 339: 104–10. 37433382 10.1016/j.jad.2023.07.042

[35] Eagly AH , Wood W. Social role theory. In Handbook of Theories of Social Psychology (eds PAM Van Lange , AW Kruglanski , ET Higgins ): 458–76. SAGE, 2012.

[36] Bonsaksen T , Thørrisen MM , Skogen JC , Aas RW. Who reported having a high-strain job, low-strain job, active job and passive job? The WIRUS Screening study. PLOS One 2019; 14: 1–13. 10.1371/journal.pone.0227336 PMC6936855 31887201

[37] Kristensen TS , Borg V , Hannerz H. Socioeconomic status and psychosocial work environment: results from a Danish national study. Scand J Public Health Suppl 2002; 59: 41–8. 12227964

[38] Shanafelt TD , Hasan O , Dyrbye LN , Sinsky C , Satele D , Sloan J , et al. Changes in burnout and satisfaction with work-life balance in physicians and the general US working population between 2011 and 2014. Mayo Clin Proc 2015; 90: 1600–13. 26653297 10.1016/j.mayocp.2015.08.023

[39] Vancappel A , Jansen E , Ouhmad N , Desmidt T , Etain B , Bergey C , et al. Psychological impact of exposure to the COVID-19 sanitary crisis on French healthcare workers: risk factors and coping strategies. Front Psychiatry 2021; 12: 1–10. 10.3389/fpsyt.2021.701127 PMC8637162 34867507

[40] Kahya E. The effects of job characteristics and working conditions on job performance. Ind Ergon 2007; 37: 515–23.

